# Attenuating Muscle Mass Loss in Critical Illness: the Role of Nutrition and Exercise

**DOI:** 10.1007/s11914-022-00746-7

**Published:** 2022-08-31

**Authors:** Lee-anne S. Chapple, Selina M. Parry, Stefan J. Schaller

**Affiliations:** 1 Intensive Care Unit, Royal Adelaide Hospital, Adelaide, South Australia Australia; 2 Adelaide Medical School, The University of Adelaide, Adelaide, South Australia Australia; 3 Centre of Research Excellence in Translating Nutritional Science to Good Health, The University of Adelaide, Adelaide, South Australia Australia; 4 Department of Physiotherapy, School of Health Sciences, The University of Melbourne, Parkville, Victoria Australia; 5 Department of Anesthesiology and Operative Intensive Care Medicine (CVK, CCM), Charité – Universitätsmedizin Berlin, corporate member of Freie Universität Berlin and Humboldt-Universität zu Berlin, Berlin, Germany; 6 Klinikum rechts der Isar, Department of Anesthesiology and Intensive Care, Technical University of Munich, School of Medicine, Munich, Germany

**Keywords:** Muscle mass, Intensive care, Recovery, Nutrition, Physical activity

## Abstract

**Purpose of Review:**

Impaired recovery following an intensive care unit (ICU) admission is thought related to muscle wasting. Nutrition and physical activity are considered potential avenues to attenuate muscle wasting. The aim of this review was to present evidence for these interventions in attenuating muscle loss or improving strength and function.

**Recent Findings:**

Randomised controlled trials on the impact of nutrition or physical activity interventions in critically ill adult patients on muscle mass, strength or function are presented. No nutrition intervention has shown an effect on strength or function, and the effect on muscle mass is conflicting. RCTs on the effect of physical activity demonstrate conflicting results; yet, there is a signal for improved strength and function with higher levels of physical activity, particularly when commenced early.

**Summary:**

Further research is needed to elucidate the impact of nutrition and physical activity on muscle mass, strength and function, particularly in combination.

## Introduction

### Muscle Loss in Critical Illness

#### Recovery from Critical Illness

Critically ill patients are the sickest in the hospital, requiring substantial medical intervention for organ support. It has been reported that 20–70% of critically ill patients have low muscle mass at baseline [1], and ICU survivorship is affected by acute and extensive muscle wasting, with up to 30% lost within the first week of an ICU admission [2••]. This muscle wasting has consequences for patient recovery, with functional disability observed in ICU survivors even five years after ICU discharge [3], leading to an increase in healthcare utilisation, delayed capacity to engage in the workforce and ultimately a loss of independence [4•, 5•]. The ability to prevent or reverse skeletal muscle loss in critically ill patients is considered paramount to recovery.

#### Reasons for Muscle Loss in ICU

The mechanisms behind the observed loss in muscle mass, strength and physical functioning are complex, likely multifactorial and still being elucidated [6]. Maintaining muscle mass is dependent on a tightly regulated equilibrium between muscle protein synthesis and muscle protein breakdown, an equilibrium that is impaired in critical illness [7]. Whilst studies of whole-body protein turnover in critical illness have shown an increase in whole-body protein synthesis when compared to healthy volunteers, this does not compensate for the higher rate of protein breakdown also observed [8•]. This catabolic state is the result of significant systemic changes, which occur at muscle, nerve, immune, metabolic and mitochondrial levels exacerbated by periods of immobility and nutrient deficits [6, 9]. In addition, critically ill patients have been shown to experience anabolic resistance, with a blunted capacity to utilise dietary protein for muscle protein synthesis [10••], and extended periods of inactivity impact on the bioenergetic level, affecting the force production capability of muscles to recover [6, 9].

### Measuring Muscle Mass, Strength and Function in Critical Illness

The measurement of skeletal muscle mass, strength and function in critical illness is met with a number of logistical challenges. Assessment of strength and function within ICU is limited by the large proportion of critically ill patients that are unable to follow commands required for active participation as a result of being intubated and ventilated or factors like delirium or fatigue in awake patients. These measures are therefore more often conducted at a later stage of the ICU/hospital admission. Given lower muscle mass and poorer muscle quality (echogenicity) have been shown to correlate with reduced strength and function [6, 11], muscle mass is frequently used as a surrogate measure that can be conducted at the bedside to measure the acute response to an intervention. Gold standard methodologies for measuring muscle mass such as dual-energy x-ray absorptiometry (DXA) or magnetic resonance imaging (MRI) are rarely feasible as patients are often too unstable to be transferred out of the ICU to undergo such measurements alongside the additional considerations of costs and radiation exposure [12]. Historically, measures of nutritional statuses, such as mid-arm muscle circumference and proved popular, though they are affected by fluid shifts in ICU [13]. More recently, the assessment of muscularity using measures of psoas muscle from computed tomography (CT) scans of the third lumbar region collected for clinical purposes have been used [14]. This technique is equally limited in application due to radiation, preventing its implementation into clinical practice. Furthermore, a range of bedside noninvasive clinical methods has been introduced into ICU to quantify muscle size including ultrasonography [15, 16] and bio-electrical impedance [17].

### Strategies to Attenuate Muscle Loss or Improve Strength or Function

Skeletal muscle is highly responsive to external stimuli such as nutrition and physical activity interventions [7]. In health, a number of nutritional strategies have been shown effective in stimulating muscle protein synthesis, including essential amino acids or their metabolites (such as leucine and HMB) [18] and higher protein doses [19]. Physical activity and dietary intake are modifiable factors associated with the risk of chronic morbidity and mortality in the general population and can positively impact on muscle mass [20, 21]. Further gains in muscle mass and strength can be induced through structured exercise such as resistance training which is needed to ‘load’ the muscle and induce a training effect [9, 22]. Accordingly, general physical activity guidelines recommend a minimum of 30 min moderate-intensity aerobic exercise on five days per week combined with at least two sessions of moderate-intensity resistance training, particularly in the older and comorbid population [23]. Nutrition and physical activity in combination may also confer greater benefits for muscle mass, strength and function than either intervention in isolation. Participation in exercise without the availability of amino acids results in rates of muscle protein breakdown that exceed rates of muscle protein synthesis leading to muscle loss [24]. Furthermore, muscle protein synthesis following amino acid provision is greater when combined with exercise than in the rested state due to an increase in both the magnitude and duration of muscle protein synthesis [25].

As ICU survivorship has improved, attention has shifted to potential strategies to improve recovery for critically ill patients. These often focus on attenuating muscle mass loss in the acute phase of illness with the aim of improving strength and function at a later time point. Given this, there has been a paradigm shift towards prioritising both nutrition and exercise interventions as part of usual ICU care practices, as reflected within recent clinical guidelines [26••, 27–29, 30••]. Consequently, there has been an exponential increase in the number of studies conducted in this important area. In this review, we discuss how nutrition and physical activity interventions may be employed in critical illness to attenuate muscle mass loss and improve strength and physical function. For the purpose of this review, we have focused on interventions commencing within the ICU setting.

## Nutrition and Physical Activity in Critical Illness

### Defining Nutrition in the ICU Setting

Critically ill patients are frequently unable to consume nutrients orally due to the need for tracheal intubation. Therefore, critical care nutrition guidelines recommend the provision of liquid nutrition via a feeding tube into the stomach – termed enteral nutrition (EN) [28, 29]. Critical care nutrition is a relatively new field of research, with a shift over the last 10 years from small physiological studies to robust large clinical trials [31]. The delivery of adequate nutrition to critically ill patients is challenging, frequently limited by extended periods of fasting and barriers to delivery caused by insulin resistance and gastrointestinal dysfunction [32•, 33]. Given this, the evidence base for nutrition recommendations, particularly those on outcomes of muscle mass, strength and function, is limited.

### Defining and Measuring Physical Activity and Exercise in the ICU Setting

Physical activity is defined as ‘any bodily movement produced by skeletal muscles that results in energy expenditure’ [34]. This encompasses all movement which may occur as part of leisure time, work or daily activities. Exercise is often used interchangeably with physical activity; however, it is important to note that exercise is a subset of physical activity [34]. Exercise is defined as ‘planned, structured, a repetitive bodily movement where the purpose is to improve or maintain physical fitness’ [34]. Physical activity and exercise can be quantified in terms of the FITT principles: frequency (i.e. how often), intensity (i.e. how hard), time (i.e. duration of individual session and overall programme length) and type of modality (i.e. cycle ergometry, functional mobility). The health benefits of regular participation in physical activity are well documented within the literature with guidelines existing for the general, older and chronic disease populations [35, 36].

Metabolic equivalent of tasks (METs) is a simple way of expressing the energy cost or intensity of physical activity [35]. The resting metabolic state is defined as one MET and refers to the amount of oxygen consumed at rest. The intensity of physical activity can be defined as low (<3.0 METs), moderate (3–5.9 METs) and vigorous (>6 METs) [35]. Within the ICU setting, patients are profoundly inactive which is in part due to the impact of their severity of illness, physiological instability, sedation, delirium and concomitant ICU life-saving treatments received [37]. External factors include ICU and hospital room designs that do not encourage awake patients to be mobile and a lack of physical therapists or nurses to perform mobilisation [38]. There is significant heterogeneity in the energy costs associated with physical activity in the ICU setting. Beach *et al.* demonstrated that some participants recorded high physical activity levels in terms of MET levels (measured using the Sensewear armband mini-fly motor sensor) even whilst sedated and not participating in rehabilitation activities. Most of these patients were septic, which can result in a hypermetabolic state and thus altered MET levels [39]. Black *et al.* assessed the oxygen costs associated with exercise interventions in mechanically ventilated ICU patients. There was significant variability in the oxygen costs of exercise between participants, which may have been influenced by factors such as the ability to actively contribute to exercise, and found that the recovery time for ~25% of sessions was longer than the total exercise duration [40•]. Dysfunctional mitochondrial functioning and regeneration capacity/production of ATP which is necessary for muscle contraction has been recognised within the ICU population [9]. More work needs to be undertaken to understand the bioenergetic costs of different types of physical activity (both in bed and out of bed) and the interplay with altered/dysfunctional mitochondrial functioning.

## Nutrition for Attenuation of Muscle Mass, Strength and Function in Critical Illness

A total of 12 RCTs of nutrition interventions in critical illness, of which two were pilot RCTs, were identified that included an outcome of muscle mass, strength or function (Table 1). Of these, the majority were conducted in Australia (*n* = 5) or Europe (*n* = 4). The interventions tested were primarily a strategy to increase nutrition overall (including calorie and protein delivery) (*n* = 7), protein delivery alone (*n* = 3) or the addition of a nutritional compound (e.g. HMB; *n* = 2). Six of the identified studies used a parenteral component to achieve greater nutrition delivery, either parenteral nutrition or intravenous amino acids alone [42–44, 46, 50, 52]. Seven studies reported an outcome related to muscle mass (including CT or ultrasound-derived muscle thickness or cross-sectional area (CSA)) [45•, 46, 47, 48•, 49•, 50, 51•], three relating to strength (handgrip strength) [46, 50, 52] and six studies relating to function (including Barthel Index, 6-min walk test and SF-36 physical component summary score) [41–44, 50, 52].

**Table 1:** Summary of randomised controlled trials of nutrition interventions on muscle mass, strength or function

Author, year, country	Population	Intervention	Control	Muscle mass, strength or functional outcome	Summary of results
Allingstrup, 2017, Denmark [41]	199 pts, MV, expected to stay >3d in ICU	Early goal-directed nutrition (based on indirect calorimetry and 24-h urinary urea)	Standard care (25 kcal/kg/day via EN)	Primary: SF-36 PCS score at 6 months	No difference in SF-36 PCS score between groups, mean, control vs intervention: 23 vs 22.9, diff (95% CI) 0.0 (−5.9–5.8), *p* = 0.99; data available for *n* = 88 vs 88 pts
Casaer, 2011, Belgium [42]	4640 pts, at nutritional risk (NRS ≥ 3)	Early PN (by day 3)	Delayed PN (by day 8)	Secondary: 6MWT iADLs	No difference in 6MWT, control vs intervention: median 277 (IQR: 210–345) vs 283 (205–336) metres, *p* = 0.57; data available for *n* = 624 vs 603 pts No difference in n (%) iADLs (control vs intervention: 779 (73.5%) vs 752 (75.5%), *p* = 0.31); data available for *n* = 1060 vs 996 pts
Doig, 2013, Australia [43]	1372 pts, contraindications to early EN, expected to stay in ICU >2 d	Early PN	Standard care	Secondary: D60 SF-36 physical function	No difference in SF-36 physical function, mean±SD: control vs intervention: 40.7±29.6 vs 42.5±30.8, diff (95% CI) 1.8 (1.85–5.52), *p* = 0.33; data available for *n* = 513 vs 524 pts
Doig, 2015, Australia [44]	474 pts, expected to stay in ICU >2 d	100 g/day IV amino acid supplementation	Standard care	Secondary: D90 SF-36 general health status and physical function	No difference in SF-36 General Health, mean±SD, control vs intervention: 52.8±25.9 vs 50.5±27.2, diff (95% CI) 2.3 (−3.1–7.7), *p* = 0.41; data available for *n* = 180 vs 192 pts No difference in SF-36 Physical Function, mean±SD, control vs intervention: 53.2±33.0 vs 47.7±33.7, diff (95% CI) 5.5 (−1.31–12.3), *p* = 0.11; data available for *n* = 180 vs 192 pts
Dresen, 2021, Germany [45•]	42 pts, MV, haemodynamically stable, expected to require >28 d organ support	1.8 g protein/kg/day	1.2 g protein/kg/day	Primary: change in ultrasound-derived QMLT from study inclusion to weeks 2 and 4	No difference in muscle loss between groups; change in mean QMLT, control vs intervention: −0.28±0.08 vs −0.15±0.08 mm; *p*=0.368
Ferrie, 2016, Australia [46]	119 pts, receiving PN, expected to receive ≥3 d of the intervention	1.2 g/kg IV amino acid	0.8 g/kg IV amino acid	Primary: handgrip strength at ICU discharge Secondary: ultrasound-derived muscle thickness of quadriceps, forearm and mid-upper arm at D7	No difference in handgrip strength, mean±SD, control vs intervention: 15.8±10.3 vs 18.5±10.4 cm; *p* = 0.054 Greater ultrasound-derived forearm muscle thickness at D7 with intervention, mean±SD, control vs intervention: 2.8±0.4 vs 3.2±0.4 cm; *p* < 0.0001
Fetterplace, 2018, Australia [47] Pilot RCT	60 pts, MV <48 h, anticipated to remain MV ≥72 h	Volume-based EN with protein supplementation	Usual care (continuous hourly rate EN)	Secondary: change in ultrasound-derived QMLT from ICU admission to discharge	Greater amelioration of QMLT loss with intervention, control vs intervention: mean diff (95% CI) 0.22 (0.06–0.38) cm, *p* = 0.01
McNelly, 2020, UK [48•]	121 pts, expected MV ≥48 h, requiring gastric EN, multiorgan failure, likely ICU stay ≥7 d and likely survival ≥10 d	Intermittent EN	Continuous EN	Primary: change in ultrasound-derived RF-CSA over 10 days	No difference in RF-CSA, mean±SD, control vs intervention: −19.8±14.2 vs −17.4±14.6 cm, diff (95% CI) −2.4 (−9.7–4.8), *p* = 0.505 Change to day 10: mean difference (95% CI): −1.1 (−6.1–4.0) %; *p* = 0.676
Nakamura, 2020, Japan [49•]	50 pts, receiving EN, haemodynamically stable	Standard EN + 3 g HMB, 14 g arginine, 14 g glutamine	Standard EN	Primary: rate of CT-derived femoral muscle volume loss from day 1 to 10	No difference in CT-derived femoral muscle loss, control vs intervention: 14.4±1.6 vs 11.4±1.6 %; *p* = 0.18
Ridley, 2018, Australia [50]	100 pts, ≥16 y, adm to ICU in previous 48–72 g, receiving MV and expected to continue until day after randomisation, central venous access, ≥1 defined organ system failure	Supplemental PN	Standard care	Secondary: ICU mobility scale (or 6MWT where possible) at hospital discharge HGS at hospital discharge MAMC at hospital discharge	No difference in ICU mobility scale, median [IQR], control vs intervention: 8 [4–10] vs 9 [5–10], *p* = 0.58; data available for *n* = 33 vs 25 pts No difference in handgrip strength, mean±SD, control vs intervention: 20±8 vs 19±13.5 kg, *p* = 0.71; data available for n=24 vs 19 pts No difference in MAMC, mean±SD, control vs intervention: 30±5 vs 30±5 cm, *p* = 0.91; data available for *n* = 25 vs 22 pts
Viana, 2021, Switzerland [51•]	30 pts, MV, likely survival ≥7 d	HMB	Placebo (maltodextrin)	Primary: magnitude of loss of ultrasound-derived quadriceps muscle CSA from day 4 to 15	No difference in muscle loss between groups; mean skeletal muscle area (control D1 114 (95% CI 43–185.8) to D14 100.4 (95% CI 32.6–168.2) cm^2^ vs intervention D1 110.5 (95% CI 43.7–177.3) to D14 99.32 (95% CI 25.7–172.92) cm^2^, *p* = 0.86
Wischmeyer, 2017, USA Pilot RCT [52]	125 pts, acute respiratory failure, expected to require MV >72, BMI <25 or ≥35	Standard EN + supplemental PN	Standard EN	Secondary: Barthel Index at hospital discharge HGS at discharge 6MWT at discharge SF-36 PCS at 3 months and 6 months	Barthel Index, mean±SD, control vs intervention: 46.5±32.1 vs 61.1±32.4, *p* = 0.08; data available for *n* = 41 vs 28 pts SF-36 PCS at 3 months, mean±SD, control vs intervention: 35.3±10.8 vs 33.3±10.1, *p* = 0.38; data available for *n* = 27 vs 22 pts SF-36 PCS at 6 months, mean±SD, control vs intervention: 35.8±11.2 vs 39.3±10.2, *p* = 0.17; data available for *n* = 30 vs 20 pts

*6MWT*, 6-minute walk test; *BMI*, body mass index; *CSA*, cross-sectional area; *CT*, computed tomography; *EN*, enteral nutrition; *D*, day; *HGS*, handgrip strength; *HMB*, B-hydroxy-B-methylbutyrate; *iADLs*, independent with activities of daily living; *IV*, intravenous; *MV*, mechanical ventilation; *PCS*, physical component summary; *PN*, parenteral nutrition; *QMLT*, quadriceps muscle layer thickness; *RCT*, randomised controlled trial; *SF-36*, Short Form 36

In 119 patients, Ferrie *et al.* delivered augmented protein intravenously compared to standard care and reported an attenuation of ultrasound-derived muscle layer thickness at day 7 with the greater protein dose (control: 2.8±0.4 vs intervention: 3.2±0.4 cm; *p* < 0.0001). This difference, however, was not sustained to ICU discharge [46]. Similarly, Fetterplace *et al.* compared augmented calorie and protein delivery to standard care in 60 patients, reporting greater amelioration of ultrasound-derived quadriceps muscle layer thickness (QMLT) loss with the intervention (mean difference (95% CI) 0.22 (0.06–0.38) cm, *p* = 0.01) [47]. These results are conflicting with more recent investigations. In 2021, Dresen *et al.* reported no difference in ultrasound-derived QMLT from study inclusion to week 2 or 4 with a higher protein dose (1.8 vs 1.2 g/kg/day [45•]); however, this study recruited patients after an extended duration of ICU stay (day 13±2 of ICU admission), and hence, the window of intervention success may have passed by this point (given muscle loss occurs early) [53]. Furthermore, McNelly *et al.* found no difference in the attenuation of rectus femoris CSA over 10 days with greater calorie/protein delivery with intermittent feeding (daily protein dose: intermittent: 63.8 (59.3–68.3) g vs control: 55.8 (49.1–62.5) g; *p* = 0.048) [48•]. Two RCTs have reported no effect on muscle mass with an intervention containing hydroxymethylbutyrate (HMB), a metabolite of leucine known to stimulate muscle protein synthesis and reduce muscle protein breakdown in health: Nakamura *et al.* reported no effect of a combined HMB/arginine/glutamine intervention on CT-derived femoral muscle volume loss [49•] and Viana *et al.* reported no difference in magnitude of the loss of ultrasound-derived quadriceps muscle CSA from day 4 to 15 [51•]. Reasons for these discrepancies in results are unclear but may be related to the timing of intervention (early versus late protein delivery) or the type of protein delivered (specific versus mixed amino acids).

No study of a nutrition intervention has been shown to be effective in improving any outcome of strength or function in critically ill patients.

## Physical Activity for Attenuation of Muscle Mass, Strength and Function in Critical Illness

Exercise interventions have been shown to be safe and feasible within the ICU setting and fall into three main modalities: neuromuscular electrical stimulation (which involves artificial stimulation of the underling muscles with surface electrodes), assistive technology such as cycle ergometry (with/without additional muscle stimulation) and functional-based strengthening and mobility training. For the purposes of this review, we have focused our reporting on cycle ergometry and functional-based mobility interventions. Recent systematic reviews have demonstrated exercise commencing in the ICU (such as mobilisation functional-based exercises) improves physical functioning at hospital discharge and reduces ICU and hospital length of stay and may improve mobility status and reduce the incidence of ICU-related weakness, muscle strength and days alive [54••, 55, 56].

A total of 28 RCTs of cycling/functional mobility in critical illness, of which three were pilot RCTs, were identified that included an outcome of muscle mass, strength or function (Tables 2 and 3). Of these, 79% of the studies were from Europe (*n* = 8), Australia (*n* = 6) or North/South America (*n* = 8). There is significant heterogeneity in terms of the modalities, frequency, timing and intensity of programmes which make it challenging to compare. This is in addition to varying trial endpoints and many lacking follow-ups beyond hospital discharge.

**Table 2:** Summary of randomised controlled trials of cycle ergometry interventions on muscle mass, strength or function

Author, year, country	Population	Timing	Intervention	Comparator	Muscle mass, strength or functional outcome	Results
Berney, 2021, Australia [57]	162 ICU patients with sepsis or systemic inflammatory response syndrome ≥48 h MV and ICU LOS ≥4 d	<72 h	60 min FES cycling >/=5 days/week until ICU discharge; single leg allocation FES cycling and other leg without FES	Usual care (respiratory and functional mobility)	Primary: quadriceps strength Secondary: MRC-SS handgrip strength PFIT-s FSS-ICU SPPB 6MWT Katz ADL RF-CSA	Primary: no significant difference between groups for quadriceps strength at hospital discharge Secondary: no significant difference between groups for any secondary measures
Burtin, 2009, Belgium [58••]	90 S/MICU patients with predicted ICU LOS >7 d	Late (>5 d after ICU admission)	Cycle ergometry 5 days/week 20 min per session individually adjusted intensity Passive 20 cycles/min or active 2× 10 min bouts increasing intensity until hospital discharge	Usual care (respiratory physiotherapy + standardised mobility of UL and LL 5 days per week) ranging from passive to active depending on the capability	Primary: 6MWD Secondary: quadriceps strength Handgrip strength Berg Balance Scale FAC SF-36 (PF domain)	Primary outcome: higher 6MWD distance in intervention at hospital discharge (196 vs 143 m, *p* < 0.05) Secondary: quadriceps strength gain higher between ICU discharge and hospital discharge in intervention (*p* < 0.01); no significant difference between groups for handgrip strength; Berg Balance Scale and FAC at ICU and hospital discharge; higher SF-36 (PF domain) scores in the intervention group at hospital discharge (21 vs 15 points, *p* < 0.01)
Eggmann, 2018, Switzerland [59]	Mixed MV ICU patients with ICU LOS ≥72 h	<48 h	5× week (with weekends as clinically indicated) up to a maximum of 3 sessions per day, endurance cycling (20 min/d at pedalling rate of 20 cycles/min) up to a max of 60 min at full resistance; resistance training for UL and LL (active assisted, weighted), 8–12 reps with 2–5 sets at 5–80% of estimated 1RM max, functional mobility tasks	Usual care (early mobility, respiratory therapy and passive/active exercises)	Primary: 6MWD and FIM Secondary: quadriceps strength Handgrip strength MRC-SS FIM TUG test SF-36	Primary: no significant difference between groups for 6MWD and FIM at hospital discharge Secondary: no significant difference in secondary outcomes
Fossat, 2018, France [60]	314 ICU patients admitted to ICU <72 h before randomisation	<48 h	1× 15 min session of cycling, 1× 50 min session/day of EMS of bilateral quads, 5× week until ICU discharge	Usual care	Primary: MRC-SS Secondary: ICU Mobility Scale Katz ADL Barthel Index SF-36 RF-CSA	No significant difference between groups in MRC-SS at ICU discharge Secondary: no significant difference between groups for any secondary measures
Gama Lordello, 2020, Brazil [61]	234 ICU cardiac surgery patients	Within 6–8 h following extubation	2× day until ICU discharge Cycle ergometry active 10 min (5 min LL, 5 min UL)	2× day 10 min of active exercises for LL and UL repeated 10×	Primary: in-hospital steps per day Secondary: mobility level in different subgroups, i.e. gender, type of surgery, pre-ICU PA	No significant difference between groups for steps per day over three days following allocated intervention Secondary: no difference in steps per day between groups
Kho, 2019, Canada [62]	66 ICU <4 d of MV and <7 d ICU LOS	<72 h	5 sessions per week of 30 min passive, to active cycling until ICU discharge + usual care	Usual care	PFIT-s	No difference between groups for PFIT-s scores at hospital discharge
Machado, 2017, Brazil [63]	38 MV ICU patients with acute respiratory failure	Median 2 d	Cycle ergometry passive to active 20 min 20 cycles/min 5× week up to ICU discharge	Conventional physiotherapy (2× 30 min daily respiratory and functional mobility)	MRC-SS	Significant improvement in MRC-SS in intervention compared to control (8.45 vs 4.18 points, *p* = 0.005)
Nickels, 2020, Australia [64]	72 mixed ICU patients expected to MV >48 h	<96 h	30 min daily in bed cycling 1× day (up to 6 days per week)	Usual care (respiratory and functional mobility)	Primary: RF-CSA at Day 10 Secondary: RF and VI thickness MRC-SS Handgrip strength FSS-ICU 6MWT ICU Mobility Scale	Primary: no significant between group differences in muscle atrophy of RF-CSA at day 10 Secondary: no significant between group differences for secondary measures

*ADL*, activities of daily living; *CSA*, cross-sectional area; *FAC*, functional ambulation category; *FES*, functional electrical stimulation; *FIM*, functional independence measure; *FSS-ICU*, functional status score in the ICU; *ICU*, intensive care unit; *LL*, lower limb; *LOS*, length of stay; *min*, minutes; *MICU*, medical ICU; *MRC-SS*, Medical Research Council sum score; *MV*, mechanical ventilation; *PA*, physical activity; *PFIT-s*, Physical Function in ICU test scored; *RF*, rectus femoris; *SICU*, Surgical ICU; *SF-36*, Short Form 36 Questionnaire; *SPPB*, Short Physical Performance Battery; *TUG test*, timed up and go test; *UL*, upper limb; *VI*, vastus intermedius; *1RM max*, one repetition maximum; *6MWT*, six-minute walk test; *6MWD*, six-minute walk distance; %, percentage

**Table 3:** Summary of randomised controlled trials of physical rehabilitation (mobility) interventions on muscle mass, strength or function

Author, year, country	Population	Timing	Intervention	Comparator	Muscle mass, strength or functional outcome	Results
Cui, 2020, China [65]	178 off-pump CABG patients aged 60 years or above	<48 h	Precision early ambulation duration and intensity determined by age-predicted maximal heart rate and V02Max. Day 1: 10 min sitting Day 2: SOOB >10 min, standing 3–5 min; walking 20m Day 3: SOOB >10 min, standing 5 min and walk minimum of 30 m. Exercises repeated up to 5 times per day	Routine ambulation – patients engaged in ambulation on day 2 or 3 after surgery	Ambulation outcome reported (but not a pre-specified primary or secondary endpoint)	Significant difference between groups for ambulation distance on day 3 (75 m vs 56 m, *p* < 0.001)
Dantas, 2012, Brazil [66]	59 ICU MV patients	Unclear (however, patients excluded if MV >7 d)	2× day, 7 times per week at a moderate intensity level in ICU	Conventional physical therapy – passive mobility of UL/LL 5× week and active assisted exercises depending on the capability	MRC-SS	Significant improvement in muscle strength over the duration of the intervention (*p* = 0.00) – however, higher baseline MRC-SS scores compared to control
Denehy, 2013, Australia [67]	150 mixed ICU patients ICU LOS >5 d	Late >5 d	Functional mobility and strengthening exercises, aerobic training beginning in ICU and continuing for 8 weeks post-hospital discharge (up to an hour) at moderate intensity	Usual care (respiratory and mobility in hospital), no outpatient service	Primary: 6MWD Secondary: TUG test SF-36 AQOL	No significant difference for 6MWD between groups at 6 months, exploratory analyses demonstrated the rate of change over time and mean between group differences in 6MWD from the first assessment greater in the intervention group NB: did not reach enrolment target of 200 Secondary: no difference between groups for secondary outcomes
Dong, 2014, China [68]	60 ICU patients with tracheal intubation or tracheostomy 48–72 h with predicted MV >7 d	48–72 h	2× day daily until hospital discharge, functional mobility tasks	Control group (unspecified)	Time to first sit out of bed in days	Faster to sit out of bed in the intervention (mean of 3.8 vs 7.3 days; *p* = 0.00)
Hickmann, 2018, Belgium [69]	19 ICU patients with septic shock <72 h	<48 h	2× 30 min session/daily for one week with 1 session of functional mobility and 1×30 min passive/active cycling	Usual care (5× week, functional mobility)	Primary: regulation of protein degradation/synthesis pathways during the first week Secondary: muscle fibre CSA Exercise-induced muscle inflammation	Primary: reduced protein degradation in the intervention group but no significant difference between groups over the first week Secondary: muscle fibre CSA preserved by exercise between days 1 and 7 (−26% in control vs 12.4% in intervention, *p* = 0.005); no significant difference between groups for exercise-induced inflammation
Hodgson, 2016, Australia Pilot RCT [70]	50 mixed ICU patients MV >48 h	<72 h	Active exercises for 1 hour per day, early goal-directed mobility focused on functional mobility	Usual care	Primary: higher maximal level and duration of activity measured using IMS Scale Secondary: PFIT-s FSS-ICU MRC-SS IADL	Higher levels of activity (mean IMS 7.3 vs 5.9; *p* = 0.05) and duration of activity in intervention (median 20 vs 7 min; *p* = 0.002) Secondary: no significant differences between groups for secondary measures
Hodgson, 2020, Australia Pilot RCT [71]	20 ICU ECMO patients	<72 h	Early goal-directed mobility	Usual care	Primary: higher maximal level and duration of activity measured using the IMS scale Secondary: Katz ADL functional independence	Primary: higher duration of mobility in the intervention (median 133 vs 27.5 min) but no difference between groups for IMS maximal score (2.67 vs 1.5 points) Secondary: between group difference in favour of early goal-directed mobility group for Katz ADL (functional independence at hospital discharge)
Kayambu, 2015, Australia Pilot RCT [72]	50 mixed CU patients with sepsis syndromes, MV >48 h	<48 h of sepsis diagnosis	1–2 × 30 min sessions/day until ICU discharge involving EMS, functional mobility and cycling	Usual care (respiratory and functional mobility)	Acute Care Index of Function at ICU discharge	No difference between groups in ACIF scores at ICU discharge
Maffei, 2017, France [73]	40 ICU liver transplant recipients	48–72 h	2× day early progressive rehabilitation involving P/AROM, functional mobility until ICU discharge	Usual care (referral to physiotherapy with 1 session per day)	Time to first mobility milestones (sitting on the edge of the bed, sitting in the chair and walking)	Patients sat on the edge of the bed sooner in the intervention group (2.6 vs 9.7 days, *p* = 0.048) No significant difference between groups for time to first sit in a chair or walking
McWilliams, 2018, UK Pilot RCT [74]	103 ICU patients MV ≥5 d	>5 d	Enhanced rehabilitation	Usual care	Manchester Mobility Score	Median time to the first mobilisation was significantly shorter in the intervention group (8 vs 10 days, *p* = 0.035) and a higher level of mobility on Manchester Mobility Score at ICU discharge (MMS 7 vs 5, *p* = 0.016)
Morris, 2016, USA [75]	300 MICU patients requiring noninvasive or invasive MV	<48 h	Standardised rehabilitation therapy involving PROM, PT and progressive resistance training, 3× sessions per day, seven days per week until hospital discharge	Usual care	Primary: hospital LOS Secondary: SPPB SF-36 (PF domain) FPI Handgrip strength HHD strength	Primary: no significant difference between groups for hospital LOS Secondary: no difference between groups for secondary outcomes except SPPB, where there was a significantly higher score for SPPB, SF-36 (PF domain) and FPI score at 6 months within the intervention group
Moss, 2016, USA [76]	120 MV (≥4 d) MICU patients	Median 8 d	Intensive rehab for 28 days (7× week in hospital and× week outpatient/home) 30 min in ICU, 60 min in ward/outpatient Programme included breathing, ROM, strength, functional mobility	Usual care (3× week focused on ROM, positioning and functional mobility) up to 28 days, no formal outpatient programme	Primary: Continuous Scale Physical Functional Performance Test Secondary: 5 times sit to stand TUG test Berg Balance Scale SF-36	Primary: no significant difference between groups for Continuous Scale Physical Performance Test scores 1-month post enrolment Secondary: no significant differences between groups for any secondary measures
Nava, 1998, Italy [77]	80 RICU COPD patients	Unspecified commenced in RICU	2× 30–45 min sessions daily of comprehensive rehab involving Steps 1 and 2: P/AROM, respiratory Rx, mobility training; step 3: respiratory muscle training 2× 10 min, cycling 1× 20 min at a workload of 15 watts and flight of 25 stairs 5×; step IV: 3 weeks 2× 30 min treadmill walking at 70% pre-exercise test score	Control group (steps 1 and 2 only)	6MWD	Significant improvement in 6MWD in intervention group at hospital discharge (*p* < 0.0001)
Nydahl, 2020, Germany [78] Cluster randomised pilot study	274 ICU patients in ICUs with no protocol for early mobility present	Median 3 d	Intervention period: goal-directed mobility plan based on ICU Mobility Scale and interprofessional rounds daily	Control period: usual care	Primary: percentage of patients with ICU Mobility Score of 3 or more	Primary: non statistically significant increase in out-of-bed mobility by 9.6%
Schaller, 2016, Germany [79••]	200 SICU patients MV <48 h and expected further MV >24 h	<48 h	Early goal-directed mobility involving daily morning ward round to set mobility goal and second goal implementation cross shifts with interprofessional communication follow-up	Usual care	Primary: SOMS level Secondary: modified FIM MRC-SS SF-36	Primary: significant differences between groups in favour of intervention for mean SOMS score Secondary: significant differences between groups for modified FIM at hospital discharge in favour of intervention; no difference between groups for MRC-SS or SF-36.
Schweickert, 2009, USA [80•]	104 pts	<48 h	Passive ROM for all limbs (10 repetitions), transitioned to active assisted and active ROM exercises, bed mobility and sitting and ADL/exercise, walking, daily basis until returned to the previous level of function or discharged from hospital	Usual care	Primary: functional independence Secondary: Barthel Index Number of functionally independent ADLs Distance walked without assistance MRC-SS Handgrip strength	Primary: greater functional independence at hospital discharge in the intervention group (59 vs 35 %, *p* = 0.02) with the faster achievement of mobility milestones (i.e. sitting, standing, marching and walking) in favour of the intervention group (*p* > 0.0001), a greater walking distance at hospital discharge Secondary: Higher Barthel Scores, a higher number of independent ADLs and greater unassisted walking distance in the intervention group at hospital discharge; non-significant difference between groups for MRC-SS and handgrip strength at hospital discharge
Seo, 2019, Korea [81]	16 ICU patients in ICU ≥5d	>5 d	Exercise group included P/AROM, resistance training, functional mobility	Cycle ergometry 5× week for 30 min until ICU discharge	MRC-SS FSS-ICU SF-36	There was a significant difference between groups for MRC-SS, FSS-ICU and SF-36 (PF domain) at ICU discharge
Schujmann, 2020, Brazil [82]	99 ICU patients scoring 100 or above on Barthel Index 2 weeks prior to ICU admission	<48 h	Combined therapy consisting of a combination of conventional therapy and a programme of early and progressive mobility. 2× day 5× week, duration ~40 min	Conventional therapy involving active assists and active mobilisation as well as bed positioning, bedside and armchair transfers and ambulation. 2× day, 5× week	Primary: Barthel Index Secondary: handgrip strength EMG of anterior tibial, medial gastroc and VL muscles TUG test Sit to stand test 2-min walk test Physical activity levels ICU Mobility Score	Higher Barthel Scores for intervention at ICU discharge (97 vs 76, *p* < 0.001) No differences between groups for handgrip strength, EMG or TUG test. Difference between groups observed for sit to stand (8 vs 5 repetitions, *p* < 0.01), 2-min walk test (*p* < 0.001) and ICU Mobility Score at ICU discharge (9.8 vs 7, *p* < 0.001). Higher levels of physical activity in the intervention (1539 steps/day vs 591 in control, *p* < 0.001).
Wright, 2017, UK [83•]	308 ICU MV ≥48 h	<72 h	90 min rehab 5× week until ICU discharge split across 2 sessions until ICU discharge	30 min rehab 5× week	Primary: SF-36 (PF domain) Secondary: modified Rivermead Mobility Index 6MWT FIM Handgrip strength	Primary: no significant difference between groups for SF-36 (PF) Secondary: no significant difference between groups for secondary measures except FIM at 3 months
Yosef Brauner, 2015, Israel [84]	18 ICU MV ≥48h and expected to remain ventilated for further 48 h		Conventional physiotherapy (more intensive 2× day) involving respiratory and functional elements – respiratory, P/AROM, functional mobility	Conventional physiotherapy	MRC-SS Handgrip strength Sitting balance	There was a significant difference in the intensive treatment group over time compared to usual care for MRC-SS (*p* = 0.029) and non-significant for handgrip and sitting balance.

*ADL*, activities of daily living; *AQOL*, Assessment of Quality of Life Questionnaire; *AROM*, active range of motion; *CSA*, cross-sectional area; *ECMO*, extra corporeal membrane oxygenation; *EMS*, electrical muscle stimulation; *FIM*, Functional Independence Measure; *FPI*, Functional Performance Inventory; *HHD*, handheld dynamometry; *IADL*, instrumented activities of daily living; *ICU*, intensive care unit; *IMS*, ICU Mobility Scale; *LOS*, length of stay; *LL*, lower limb; *MICU*, medical ICU; *min*, minutes; *MRC-SS*, Medical Research Council sum score; *MV*, mechanical ventilation; *PFIT-s*, Physical Function in ICU test scored; *PROM*, passive range of motion; *PT*, physiotherapy; *Rx*, treatment; *SF-36*, Short Form 36 Questionnaire; *SOM*, Surgical Optimal Mobility Scale; *SPPB*, short physical performance battery; *TUG test*, timed up and go test; *UL*, upper limb; *6MWD*, six-minute walk distance; *%*, percentage

Exercise can be considered a drug as it causes a range of beneficial effects for health, as do pharmacological interventions [85]. Drug trials adhere to rigorous testing processes to determine the minimum effective dose, with titration up to a maximum dose level beyond which the adverse effects of the drug outweigh the benefits. Exercise trials have traditionally not undergone the same scrutiny as drug trials. Currently, the exercise dose that a patient receives is poorly described and articulated within ICU trials. This is in part due to the lack of consistency in defining the ‘dosage’ of interventions and reporting of the actual versus planned intervention delivery. Recently within the stroke literature, a dose articulation framework has been developed to improve the rigour in exercise dosage reporting which is also applicable to the ICU setting [86•]. Scheffenbichler *et al*. used a Mobilisation Quantification Score to address the problem of dose [87•]. Within exercise dosage, we need comprehensive reporting of what is planned and then what was delivered with consideration of the FITT principles: frequency, intensity, time (individual session duration and overall programme length) and type of activities (including individual tasks, task duration) [86•].

### Timing and Duration of Intervention

It appears that the greatest benefit may be observed in trials commencing within the first 72 hours of ICU admission with trials demonstrating higher muscle strength, functional independence, higher level of mobility including distance able to be walked and earlier attainment of mobility milestones at hospital discharge (Table 3). It also appears that rehabilitation delivered less than 5 days per week may be less effective [55, 79••, 88]. The length of the ICU-based exercise programmes may be another confounder. Numerous trials have had a median of 3–7 sessions delivered (often over 7–10 days) which may be too short an intervention period to induce changes in muscle mass, strength and function.

### Frequency/Intensity

Achievement of higher levels of mobility has been related to better physical recovery outcomes for ICU survivors [70, 79••, 87•, 89•]. Conflicting evidence exists with regards to the increasing frequency of sessions, with several studies demonstrating 2× sessions per day resulted in earlier attainment of mobility milestones and improved strength [66, 68, 69, 73, 84]. This contrasts with a recently published secondary analysis of a prospective study of 186 ICU patients which found that increasing the number of mobility sessions did not independently influence health status 6 months post-ICU admission. It is important to note that there was variability in the amount of active mobilisation sessions performed in ICU, with 19% of the cohort performing less than one session per week and just under half completing a mobility session every 1–3 days with less than 5% completing more than one session per day. More research is required to elucidate the prescription parameters in terms of intensity, frequency and duration which may result in the greatest long-term benefits for ICU patients [61, 89•]. Several trials have attempted to provide higher programme intensities but failed to implement these targets [59, 83•]. The discrepancy between planned and actual therapy delivery in these trials has occurred due to patient- and setting-related barriers (e.g. participant fatigue, sedation) [59, 83•] as well as logistic challenges with missed physiotherapy visits due to weekends, medical procedures and/or physiological instability [59, 83•]. It may be that the field has overestimated how much patients may be able to achieve in the early ICU period, as it is likely that the muscle fatigue threshold required for a training response is lower in critical illness, particularly in the context of impaired muscle/nerve functioning [54••]. More work needs to be undertaken to understand the impact of fatigue and to develop personalised approaches to prescribing exercise doses within the ICU population.

### Exercise Mode

Functional-based movement is the most used exercise intervention within the ICU setting and often involves sitting, standing, walking and resistance-based exercises (Table 3). The importance of goal-directed early mobility has been emphasised in recent trials in terms of interprofessional communication and optimising patient status (in terms of sedation, pain, delirium) to achieve a target mobility level [70, 79••]. Several studies have proven that mobility can improve strength and physical functioning and impact on other important outcomes such as delirium and length of stay. There has been growing interest in the last 10 years in non-volitional exercise interventions which may enable earlier targeted optimisation of muscle mass and strength due to the awareness of muscle wasting occurring early and rapidly and the delay until patients are alert and able to engage in functional-based exercises [90]. Electrical muscle stimulation which involves artificial stimulation of the muscle using transcutaneous electrodes placed over the skin is one promising modality [56]. There is conflicting evidence; however, some studies have demonstrated the preservation of muscle mass and strength within the ICU setting [91•, 92]. The optimal stimulation parameters and impact on long-term outcomes need to be determined as well as the patient subgroups who may be of most benefit. Cycle ergometry is another attractive intervention which can be utilised passively (without patient effort whilst in a coma) and actively with increasing resistance. Burtin *et al.* conducted the first RCT of cycle ergometry compared to usual care in the ICU which found significant improvements in exercise capacity as measured by the six-minute walk distance (196 vs 143 m, *p* < 0.05), and quadriceps force improved more between ICU and hospital discharge in the treatment group (1.83 vs 2.37 N/kg (intervention), 1.86 vs 2.03 N/kg, *p* < 0.01) [58••]. Subsequent cycle trials have found conflicting results with heterogeneity in outcomes measured, timing of intervention and dosage parameters used, making it difficult to make comparisons [59–64] (Table 2).

## Nutrition and Physical Activity – a Combined Intervention?

As we understand that the combination of amino acid administration and exercise has a synergistic effect on stimulating muscle protein synthesis in health [24, 25], we also need to understand the mutual benefit of combined interventions of nutrition and exercise which may augment gains in muscle mass, strength and physical functioning in critical illness. Only two RCTs in critical illness have been published that explore the combination of nutrition and physical activity interventions on outcomes of muscle mass, strength or function (Table 4). De Azevedo *et al.* randomised 181 patients to receive either nutrition guided by indirect calorimetry with augmented protein delivery (including via supplemental parenteral nutrition) and twice daily cycle ergometry exercise or standard care, reporting improved function at 3 months and 6 months with the intervention when using the SF-36 Physical Component Summary score, but no difference in handgrip strength between groups [94••]. Zhou *et al.* randomised 150 patients into one of three study arms: standard care versus early mobilisation versus early mobilisation plus early nutrition (within 48 hours of ICU admission) [93••]. They reported reduced ICU-acquired weakness and improved functional status using Barthel Index with both interventions compared to standard care.

**Table 4:** Summary of randomised controlled trials of combined nutrition and physical activity interventions on muscle mass, strength or function

Author, year, country	Population	Timing	Intervention	Comparator	Muscle mass, strength or functional outcome	Results
Zhou, 2022, China [93••]	150 pts, adm to ICU for the first time, expected ICU stay ≥72 h, conscious enough to respond (*n* = 50 pts per group)	<24 h	2 intervention arms: EM: early mobilisation (20-30 min 2×/day within 24 h) EMN: early mobilisation as per EM group + early nutrition (within 48 h of ICU adm)	Standard care: routine rehabilitation exercise and nutrition support	Primary: ICU-AW (MRC sum score <48) at ICU discharge Secondary: muscle strength from MRC sum score Barthel Index	Lower rates of ICU-AW in intervention groups, mean (95% CI), control vs intervention: 16 (7.2–29) % vs EM: 2 (0.1–10.6) % vs EMN: 2 (0.1–10.6) %; *p* = 0.005 MRC sum score did not differ between groups, mean (95% CI), control vs intervention: 60 (56.5–60) % vs EM: 60 (59.8–60) % vs EMN: 60 (60–60) %; *p* = 0.225 Improved Barthel Index with interventions mean (95% CI), control vs intervention: 57.5 (38.8–70) % vs EM: 70 (50–81.3) % vs EMN: 70 (55–80) %; *p* = 0.008
De Azevedo, 2021, Brazil [94••]	181 pts, MV, expected ICU stay >3 d		Nutrition guided by indirect calorimetry + high protein intake (including supp PN), cycle ergometry exercise 2×/d	Routine physiotherapy, standard nutrition provision	Primary: SF-36 PCS at 3 months and 6 months Secondary: ICU-AW defined by HGS ICU discharge	Better SF-36 PCS at 3 monts with the intervention, median (IQR), control vs intervention: 0.00 (0.00–37.0) vs 24.4 (0.00–49.12); *p* = 0.01 Better SF-36 PCS at 6 months with the intervention, median (IQR), control vs intervention: 0.00 (0.00–55.1) vs 33.63 (0.00–71.61); *p* = 0.01 No difference in HGS ICU-AW, *n* (%), control vs intervention: 26 (46.4%) vs 16 (28.5%); *p* = 0.05

## Future Directions

As this research field advances, we will continue to see a greater focus on combined exercise and nutrition therapies, with a number of clinical trials registered on this topic [95]. The ICU population is a highly heterogeneous population in terms of admission diagnoses and comorbid health statuses. Pre-ICU health factors such as comorbidities, age, sex and baseline nutritional status are likely to impact the response to exercise and nutrition, as well as the post-ICU recovery trajectory [96]. Therefore, a personalised approach to nutrition and exercise delivery may be needed with the identification of subgroups who may respond to different therapies and dosage levels. Greater articulation of the planned intervention delivery and actual delivery against intervention reporting frameworks are required. The separation between intervention and usual care also needs to be clearly documented, particularly as usual care nutrition delivery and mobility practices continue to evolve.

A wide range of outcome measures relating to muscle mass, strength and function are currently reported in RCTs of nutrition and physical activity interventions, restricting synthesis and interpretation of results. Core outcome sets have been published for long-term ICU recovery follow-up [97•, 98] and are being developed for physical rehabilitation [99] and nutrition fields [100], which need to be adopted in order to improve comparability across future trials.

Further work is also likely to focus on the post-ICU phase, on the premise that nutrition and physical activity interventions of a sustained duration, throughout the hospital admission, are likely to be required. Whilst observational data report suboptimal nutrition intake [101, 102] and reduced physical activity [37, 39, 103] in ICU survivors, studies conducted throughout the hospital admission are lacking.

## Conclusions

Overall, few studies have quantified the effect of a nutrition intervention on muscle mass, strength or function in critical illness, and both the intervention and the outcome technique used vary greatly, limiting interpretation. Few studies have quantified the role of nutrition on muscle mass, strength or function, with conflicting results, particularly in relation to the role of augmented protein dose on attenuation of muscle mass loss. The time at which physical activity interventions are commenced appears to be important, with the greatest benefit seen when intervening within the first 72 hours and ensuring a sufficient intensity of exercise. However, more work needs to be undertaken to articulate the exercise dose considerations and identify the responders who may benefit from different types of personalised approaches to optimising physical activity levels to preserve muscle mass and strength and optimising physical functioning.

## References

[1] Moisey LL, Mourtzakis M, Cotton BA, Premji T, Heyland DK, Wade CE (2013). Skeletal muscle predicts ventilator-free days, ICU-free days, and mortality in elderly ICU patients. Critical Care (London, England).

[2.••] Puthucheary ZA, Rawal J, McPhail M, Connolly B, Ratnayake G, Chan P (2013). Acute skeletal muscle wasting in critical illness. JAMA.

[3] Herridge MS, Cheung AM, Tansey CM, Matte-Martyn A, Diaz-Granados N, Al-Saidi F (2003). One-year outcomes in survivors of the acute respiratory distress syndrome. N Engl J Med.

[4.•] Deane AM, Little L, Bellomo R, Chapman MJ, Davies AR, Ferrie S (2020). Outcomes six months after delivering 100% or 70% of enteral calorie requirements during critical illness (TARGET). A randomized controlled trial. Am J Respir Crit Care Med.

[5.•] Parry SM, Nalamalapu SR, Nunna K, Rabiee A, Friedman LA, Colantuoni E (2021). Six-minute walk distance after critical illness: a systematic review and meta-analysis. J Intensive Care Med.

[6] Batt J, Herridge MS, Dos Santos CC (2019). From skeletal muscle weakness to functional outcomes following critical illness: a translational biology perspective. Thorax.

[7] Parry SM, Puthucheary ZA (2015). The impact of extended bed rest on the musculoskeletal system in the critical care environment. Extrem Physiol Med.

[8.•] Liebau F, Deane AM, Rooyackers O (2021). Protein absorption and kinetics in critical illness. Curr Opin Clin Nutr Metab Care.

[9] Bear DE, Parry SM, Puthucheary ZA (2018). Can the critically ill patient generate sufficient energy to facilitate exercise in the ICU?. Curr Opin Clin Nutr Metab Care.

[10] •• Chapple LS, Kouw IWK, Summers MJ, Weinel LM, Gluck S, Raith E, et al. Muscle protein synthesis following protein administration in critical illness. Am J Respir Crit Care Med. 2022. 10.1164/rccm.202112-2780OC**Using comprehensive tracer methodology, this study reported skeletal muscle protein synthesis following intraduodenally delivered dietary protein is impaired in critical illness, despite near normal protein digestion and amino acid absorption.**

[11] Hayashida I, Tanimoto Y, Takahashi Y, Kusabiraki T, Tamaki J (2014). Correlation between muscle strength and muscle mass, and their association with walking speed, in community-dwelling elderly Japanese individuals. PLoS One.

[12] Looijaard W, Molinger J, Weijs PJM (2018). Measuring and monitoring lean body mass in critical illness. Curr Opin Crit Care.

[13] Ismael S, Savalle M, Trivin C, Gillaizeau F, D'Auzac C, Faisy C (2014). The consequences of sudden fluid shifts on body composition in critically ill patients. Crit Care.

[14] Looijaard WG, Dekker IM, Stapel SN, Girbes AR, Twisk JW, Oudemans-van Straaten HM (2016). Skeletal muscle quality as assessed by CT-derived skeletal muscle density is associated with 6-month mortality in mechanically ventilated critically ill patients. Crit Care.

[15] Mourtzakis M, Parry S, Connolly B, Puthucheary Z (2017). Skeletal muscle ultrasound in critical care: a tool in need of translation. Ann Am Thorac Soc.

[16] Klawitter F, Walter U, Patejdl R, Endler J, Reuter DA, Ehler J (2022). Sonographic evaluation of muscle echogenicity for the detection of intensive care unit-acquired weakness: a pilot single-center prospective cohort study. Diagnostics (Basel).

[17] Baldwin CE, Fetterplace K, Beach L, Kayambu G, Paratz J, Earthman C, et al. Early Detection of muscle weakness and functional limitations in the critically ill: a retrospective evaluation of bioimpedance spectroscopy. JPEN J Parenter Enteral Nutr. 2019. 10.1002/jpen.1719 31583738

[18] Holecek M, Vodenicarovova M (2019). Effects of beta-hydroxy-beta-methylbutyrate supplementation on skeletal muscle in healthy and cirrhotic rats. Int J Exp Pathol.

[19] Cuthbertson D, Smith K, Babraj J, Leese G, Waddell T, Atherton P (2005). Anabolic signaling deficits underlie amino acid resistance of wasting, aging muscle. FASEB J.

[20] Oikawa SY, Holloway TM, Phillips SM (2019). The impact of step reduction on muscle health in aging: protein and exercise as countermeasures. Front Nutr.

[21] Nishiguchi S, Yamada M, Kajiwara Y, Sonoda T, Yoshimura K, Kayama H (2014). Effect of physical activity at midlife on skeletal muscle mass in old age in community-dwelling older women: a cross-sectional study. J Clin Gerontol Geriatr.

[22] Dempsey PC, Friedenreich CM, Leitzmann MF, Buman MP, Lambert E, Willumsen J (2021). Global public health guidelines on physical activity and sedentary behavior for people living with chronic conditions: a call to action. J Phys Act Health.

[23] Garber CE, Blissmer B, Deschenes MR, Franklin BA, Lamonte MJ, Lee IM (2011). American College of Sports Medicine position stand. Quantity and quality of exercise for developing and maintaining cardiorespiratory, musculoskeletal, and neuromotor fitness in apparently healthy adults: guidance for prescribing exercise. Med Sci Sports Exerc.

[24] Biolo G, Maggi SP, Williams BD, Tipton KD, Wolfe RR (1995). Increased rates of muscle protein turnover and amino acid transport after resistance exercise in humans. Am J Phys.

[25] Pennings B, Koopman R, Beelen M, Senden JM, Saris WH, van Loon LJ (2011). Exercising before protein intake allows for greater use of dietary protein-derived amino acids for de novo muscle protein synthesis in both young and elderly men. Am J Clin Nutr.

[26.••] Devlin JW, Skrobik Y, Gelinas C, Needham DM, Slooter AJC, Pandharipande PP (2018). Clinical practice guidelines for the prevention and management of pain, agitation/sedation, delirium, immobility, and sleep disruption in adult patients in the ICU. Crit Care Med.

[27] Lang JK, Paykel MS, Haines KJ, Hodgson CL (2020). Clinical practice guidelines for early mobilization in the ICU: a systematic review. Crit Care Med.

[28] McClave SA, Taylor BE, Martindale RG, Warren MM, Johnson DR, Braunschweig C (2016). Guidelines for the provision and assessment of nutrition support therapy in the adult critically ill patient: Society of Critical Care Medicine (SCCM) and American Society for Parenteral and Enteral Nutrition (A.S.P.E.N.). JPEN J Parenter Enteral Nutr.

[29] Singer P, Blaser AR, Berger MM, Alhazzani W, Calder PC, Casaer MP (2019). ESPEN guideline on clinical nutrition in the intensive care unit. Clin Nutr.

[30.••] Compher C, Bingham AL, McCall M, Patel J, Rice TW, Braunschweig C (2022). Guidelines for the provision of nutrition support therapy in the adult critically ill patient: The American Society for Parenteral and Enteral Nutrition. JPEN J Parenter Enteral Nutr.

[31] Chapple LS, Ridley EJ, Chapman MJ (2020). Trial design in critical care nutrition: the past, present and future. Nutrients.

[32.•] Reintam Blaser A, Preiser JC, Fruhwald S, Wilmer A, Wernerman J, Benstoem C (2020). Gastrointestinal dysfunction in the critically ill: a systematic scoping review and research agenda proposed by the Section of Metabolism, Endocrinology and Nutrition of the European Society of Intensive Care Medicine. Crit Care.

[33] Carbon NM, Engelhardt LJ, Wollersheim T, Grunow JJ, Spies CD, Mardian S (2022). Impact of protocol-based physiotherapy on insulin sensitivity and peripheral glucose metabolism in critically ill patients. J Cachexia Sarcopenia Muscle.

[34] Caspersen C (1985). Physical activity, exercise and physical fitness: definitions and distinctions for health related research. Public Health Rep.

[35] Piercy KL, Troiano RP, Ballard RM, Carlson SA, Fulton JE, Galuska DA (2018). The physical activity guidelines for Americans. JAMA.

[36] Sparling PB, Howard BJ, Dunstan DW, Owen N (2015). Recommendations for physical activity in older adults. BMJ.

[37] Parry SM, Knight LD, Connolly B, Baldwin C, Puthucheary Z, Morris P (2017). Factors influencing physical activity and rehabilitation in survivors of critical illness: a systematic review of quantitative and qualitative studies. Intensive Care Med.

[38] Hermes C, Nydahl P, Blobner M, Dubb R, Filipovic S, Kaltwasser A (2020). Assessment of mobilization capacity in 10 different ICU scenarios by different professions. PLoS One.

[39] Beach LJ, Fetterplace K, Edbrooke L, Parry SM, Curtis R, Rechnitzer T (2017). Measurement of physical activity levels in the intensive care unit and functional outcomes: an observational study. J Crit Care.

[40.•] Black C, Grocott M, Singer M (2020). The oxygen cost of rehabilitation interventions in mechanically ventilated patients: an observational study. Physiotherapy.

[41] Allingstrup MJ, Kondrup J, Wiis J, Claudius C, Pedersen UG, Hein-Rasmussen R (2017). Early goal-directed nutrition versus standard of care in adult intensive care patients: the single-centre, randomised, outcome assessor-blinded EAT-ICU trial. Intensive Care Med.

[42] Casaer MP, Mesotten D, Hermans G, Wouters PJ, Schetz M, Meyfroidt G (2011). Early versus late parenteral nutrition in critically ill adults. N Engl J Med.

[43] Doig GS, Simpson F, Sweetman EA, Finfer SR, Cooper DJ, Heighes PT (2013). Early parenteral nutrition in critically ill patients with short-term relative contraindications to early enteral nutrition: a randomized controlled trial. JAMA.

[44] Doig GS, Simpson F, Bellomo R, Heighes PT, Sweetman EA, Chesher D (2015). Intravenous amino acid therapy for kidney function in critically ill patients: a randomized controlled trial. Intensive Care Med.

[45.•] Dresen E, Weissbrich C, Fimmers R, Putensen C, Stehle P (2021). Medical high-protein nutrition therapy and loss of muscle mass in adult ICU patients: a randomized controlled trial. Clin Nutr.

[46] Ferrie S, Allman-Farinelli M, Daley M, Smith K (2016). Protein requirements in the critically ill: a randomized controlled trial using parenteral nutrition. JPEN. J Parenter Enter Nutr.

[47] Fetterplace K, Deane AM, Tierney A, Beach LJ, Knight LD, Presneill J, et al. Targeted full energy and protein delivery in critically ill patients: a pilot randomized controlled trial (FEED Trial). JPEN: J Parenter Enteral Nutr. 2018. 10.1002/jpen.1166 29701878

[48.•] McNelly AS, Bear DE, Connolly BA, Arbane G, Allum L, Tarbhai A (2020). Effect of intermittent or continuous feed on muscle wasting in critical illness: a phase 2 clinical trial. Chest.

[49.•] Nakamura K, Kihata A, Naraba H, Kanda N, Takahashi Y, Sonoo T (2020). beta-Hydroxy-beta-methylbutyrate, arginine, and glutamine complex on muscle volume loss in critically ill patients: a randomized control trial. JPEN J Parenter Enteral Nutr.

[50] Ridley EJ, Davies AR, Parke R, Bailey M, McArthur C, Gillanders L (2018). Supplemental parenteral nutrition versus usual care in critically ill adults: a pilot randomized controlled study. Crit Care.

[51.•] Viana MV, Becce F, Pantet O, Schmidt S, Bagnoud G, Thaden JJ (2021). Impact of beta-hydroxy-beta-methylbutyrate (HMB) on muscle loss and protein metabolism in critically ill patients: a RCT. Clin Nutr.

[52] Wischmeyer PE, Hasselmann M, Kummerlen C, Kozar R, Kutsogiannis DJ, Karvellas CJ (2017). A randomized trial of supplemental parenteral nutrition in underweight and overweight critically ill patients: the TOP-UP pilot trial. Crit Care.

[53] Gamrin-Gripenberg L, Sundstrom-Rehal M, Olsson D, Grip J, Wernerman J, Rooyackers O (2018). An attenuated rate of leg muscle protein depletion and leg free amino acid efflux over time is seen in ICU long-stayers. Crit Care.

[54.••] Wang YT, Lang JK, Haines KJ, Skinner EH, Haines TP (2022). Physical rehabilitation in the ICU: a systematic review and meta-analysis. Crit Care Med.

[55] Tipping CJ, Harrold M, Holland A, Romero L, Nisbet T, Hodgson CL (2017). The effects of active mobilisation and rehabilitation in ICU on mortality and function: a systematic review. Intensive Care Med.

[56] Waldauf P, Jiroutkova K, Krajcova A, Puthucheary Z, Duska F (2020). Effects of rehabilitation interventions on clinical outcomes in critically ill patients: systematic review and meta-analysis of randomized controlled trials. Crit Care Med.

[57] Berney S, Hopkins RO, Rose JW, Koopman R, Puthucheary Z, Pastva A (2021). Functional electrical stimulation in-bed cycle ergometry in mechanically ventilated patients: a multicentre randomised controlled trial. Thorax.

[58.••] Burtin C, Clerckx B, Robbeets C, Ferdinande P, Langer D, Troosters T (2009). Early exercise in critically ill patients enhances short-term functional recovery. Crit Care Med.

[59] Eggmann S, Verra ML, Luder G, Takala J, Jakob SM (2018). Effects of early, combined endurance and resistance training in mechanically ventilated, critically ill patients: a randomised controlled trial. PLoS One.

[60] Fossat G, Baudin F, Courtes L, Bobet S, Dupont A, Bretagnol A (2018). Effect of in-bed leg cycling and electrical stimulation of the quadriceps on global muscle strength in critically ill adults: a randomized clinical trial. JAMA.

[61] Gama Lordello GG, Goncalves Gama GG, Lago Rosier G, Viana P, Correia LC, Fonteles Ritt LE (2020). Effects of cycle ergometer use in early mobilization following cardiac surgery: a randomized controlled trial. Clin Rehabil.

[62] Kho ME, Molloy AJ, Clarke FJ, Reid JC, Herridge MS, Karachi T (2019). Multicentre pilot randomised clinical trial of early in-bed cycle ergometry with ventilated patients. BMJ Open Respir Res.

[63] Machado ADS, Pires-Neto RC, Carvalho MTX, Soares JC, Cardoso DM, Albuquerque IM (2017). Effects that passive cycling exercise have on muscle strength, duration of mechanical ventilation, and length of hospital stay in critically ill patients: a randomized clinical trial. J Bras Pneumol.

[64] Nickels MR, Aitken LM, Barnett AG, Walsham J, King S, Gale NE (2020). Effect of in-bed cycling on acute muscle wasting in critically ill adults: a randomised clinical trial. J Crit Care.

[65] Cui Z, Li N, Gao C, Fan Y, Zhuang X, Liu J (2020). Precision implementation of early ambulation in elderly patients undergoing off-pump coronary artery bypass graft surgery: a randomized-controlled clinical trial. BMC Geriatr.

[66] Dantas CM, Silva PF, Siqueira FH, Pinto RM, Matias S, Maciel C (2012). Influence of early mobilization on respiratory and peripheral muscle strength in critically ill patients. Rev Bras Ter Intensiva.

[67] Denehy L, Skinner EH, Edbrooke L, Haines K, Warrillow S, Hawthorne G (2013). Exercise rehabilitation for patients with critical illness: a randomized controlled trial with 12 months of follow-up. Crit Care.

[68] Dong ZH, Yu BX, Sun YB, Fang W, Li L (2014). Effects of early rehabilitation therapy on patients with mechanical ventilation. World J Emerg Med.

[69] Hickmann CE, Castanares-Zapatero D, Deldicque L, Van den Bergh P, Caty G, Robert A (2018). Impact of very early physical therapy during septic shock on skeletal muscle: a randomized controlled trial. Crit Care Med.

[70] Hodgson CL, Bailey M, Bellomo R, Berney S, Buhr H, Denehy L (2016). A binational multicenter pilot feasibility randomized controlled trial of early goal-directed mobilization in the ICU. Crit Care Med.

[71] ECMO-PT Study Investigators and International ECMO Network (2020). Early mobilisation during extracorporeal membrane oxygenation was safe and feasible: a pilot randomised controlled trial. Intensive Care Med.

[72] Kayambu G, Boots R, Paratz J (2015). Early physical rehabilitation in intensive care patients with sepsis syndromes: a pilot randomised controlled trial. Intensive Care Med.

[73] Maffei P, Wiramus S, Bensoussan L, Bienvenu L, Haddad E, Morange S (2017). Intensive early rehabilitation in the intensive care unit for liver transplant recipients: a randomized controlled trial. Arch Phys Med Rehabil.

[74] McWilliams D, Jones C, Atkins G, Hodson J, Whitehouse T, Veenith T (2018). Earlier and enhanced rehabilitation of mechanically ventilated patients in critical care: a feasibility randomised controlled trial. J Crit Care.

[75] Morris PE, Berry MJ, Files DC, Thompson JC, Hauser J, Flores L (2016). Standardized rehabilitation and hospital length of stay among patients with acute respiratory failure: a randomized clinical trial. JAMA.

[76] Moss M, Nordon-Craft A, Malone D, Van Pelt D, Frankel SK, Warner ML (2016). A randomized trial of an intensive physical therapy program for patients with acute respiratory failure. Am J Respir Crit Care Med.

[77] Nava S (1998). Rehabilitation of patients admitted to a respiratory intensive care unit. Arch Phys Med Rehabil.

[78] Nydahl P, Gunther U, Diers A, Hesse S, Kerschensteiner C, Klarmann S (2020). PROtocol-based MObilizaTION on intensive care units: stepped-wedge, cluster-randomized pilot study (Pro-Motion). Nurs Crit Care.

[79.••] Schaller SJ, Anstey M, Blobner M, Edrich T, Grabitz SD, Gradwohl-Matis I (2016). Early, goal-directed mobilisation in the surgical intensive care unit: a randomised controlled trial. Lancet.

[80.•] Schweickert WD, Pohlman MC, Pohlman AS, Nigos C, Pawlik AJ, Esbrook CL (2009). Early physical and occupational therapy in mechanically ventilated, critically ill patients: a randomised controlled trial. Lancet.

[81] Seo B, Shin W-S (2019). Effects of functional training on strength, function level, and quality of life of persons in intensive care units. Phys Ther Rehabil Sci.

[82] Schujmann DS, Teixeira Gomes T, Lunardi AC, Zoccoler Lamano M, Fragoso A, Pimentel M (2020). Impact of a progressive mobility program on the functional status, respiratory, and muscular systems of ICU patients: a randomized and controlled trial. Crit Care Med.

[83.•] Wright SE, Thomas K, Watson G, Baker C, Bryant A, Chadwick TJ (2018). Intensive versus standard physical rehabilitation therapy in the critically ill (EPICC): a multicentre, parallel-group, randomised controlled trial. Thorax.

[84] Yosef-Brauner O, Adi N, Ben Shahar T, Yehezkel E, Carmeli E (2015). Effect of physical therapy on muscle strength, respiratory muscles and functional parameters in patients with intensive care unit-acquired weakness. Clin Respir J.

[85] Vina J, Sanchis-Gomar F, Martinez-Bello V, Gomez-Cabrera MC (2012). Exercise acts as a drug; the pharmacological benefits of exercise. Br J Pharmacol.

[86.•] Hayward KS, Churilov L, Dalton EJ, Brodtmann A, Campbell BCV, Copland D (2021). Advancing stroke recovery through improved articulation of nonpharmacological intervention dose. Stroke.

[87.•] Scheffenbichler FT, Teja B, Wongtangman K, Mazwi N, Waak K, Schaller SJ (2021). Effects of the level and duration of mobilization therapy in the surgical ICU on the loss of the ability to live independently: an international prospective cohort study. Crit Care Med.

[88] Ding N, Zhang Z, Zhang C, Yao L, Yang L, Jiang B (2019). What is the optimum time for initiation of early mobilization in mechanically ventilated patients? A network meta-analysis. PLoS One.

[89.•] Paton M, Lane R, Paul E, Cuthburtson GA, Hodgson CL (2021). Mobilization during critical illness: a higher level of mobilization improves health status at 6 months, a secondary analysis of a prospective cohort study. Crit Care Med.

[90] Schaller SJ, Scheffenbichler FT, Bose S, Mazwi N, Deng H, Krebs F (2019). Influence of the initial level of consciousness on early, goal-directed mobilization: a post hoc analysis. Intensive Care Med.

[91.•] Parry SM, Berney S, Granger CL, Koopman R, El-Ansary D, Denehy L (2013). Electrical muscle stimulation in the intensive care setting: a systematic review. Crit Care Med.

[92] Burke D, Gorman E, Stokes D, Lennon O (2016). An evaluation of neuromuscular electrical stimulation in critical care using the ICF framework: a systematic review and meta-analysis. Clin Respir J.

[93.••] Zhou W, Yu L, Fan Y, Shi B, Wang X, Chen T (2022). Effect of early mobilization combined with early nutrition on acquired weakness in critically ill patients (EMAS): a dual-center, randomized controlled trial. PLoS One.

[94.••] de Azevedo JRA, Lima HCM, Frota P, Nogueira I, de Souza SC, Fernandes EAA (2021). High-protein intake and early exercise in adult intensive care patients: a prospective, randomized controlled trial to evaluate the impact on functional outcomes. BMC Anesthesiol.

[95] Heyland DK, Day A, Clarke GJ, Hough CT, Files DC, Mourtzakis M (2019). Nutrition and exercise in critical illness trial (NEXIS trial): a protocol of a multicentred, randomised controlled trial of combined cycle ergometry and amino acid supplementation commenced early during critical illness. BMJ Open.

[96] Amundadottir OR, Jonasdottir RJ, Sigvaldason K, Jonsdottir H, Moller AD, Dean E (2020). Predictive variables for poor long-term physical recovery after intensive care unit stay: an exploratory study. Acta Anaesthesiol Scand.

[97.•] Needham DM, Sepulveda KA, Dinglas VD, Chessare CM, Friedman LA, Bingham CO (2017). Core outcome measures for clinical research in acute respiratory failure survivors. An International Modified Delphi Consensus Study. Am J Respir Crit Care Med.

[98] Spies CD, Krampe H, Paul N, Denke C, Kiselev J, Piper SK (2021). Instruments to measure outcomes of post-intensive care syndrome in outpatient care settings – results of an expert consensus and feasibility field test. J Intensive Care Soc.

[99] Connolly B, Denehy L, Hart N, Pattison N, Williamson P, Blackwood B (2018). Physical Rehabilitation Core Outcomes In Critical illness (PRACTICE): protocol for development of a core outcome set. Trials.

[100] Puthucheary Z, Davies T. Core outcome measures for clinical effectiveness trials of nutritional and metabolic interventions in critical illness: an International Modified Delphi Consensus Study Evaluation (CONCISE) 2022 [Available from: https://www.comet-initiative.org/Studies/Details/1838. 10.1186/s13054-022-04113-x PMC9357332 35933433

[101] Chapple LS, Deane AM, Heyland DK, Lange K, Kranz AJ, Williams LT, et al. Energy and protein deficits throughout hospitalization in patients admitted with a traumatic brain injury. Clin Nutr. 2016. 10.1016/j.clnu.2016.02.009 26949198

[102] Ridley EJ, Parke RL, Davies AR, Bailey M, Hodgson C, Deane AM (2019). What happens to nutrition intake in the post-intensive care unit hospitalization period? An observational cohort study in critically ill adults. JPEN J Parenter Enteral Nutr.

[103] Gupta P, Martin JL, Needham DM, Vangala S, Colantuoni E, Kamdar BB (2020). Use of actigraphy to characterize inactivity and activity in patients in a medical ICU. Heart Lung.

